# The association of *TMPRSS6* gene polymorphism with iron status in Egyptian children (a pilot study)

**DOI:** 10.1186/s12887-024-04573-w

**Published:** 2024-02-10

**Authors:** Hanan M. Hamed, Eman El Bostany, Ayat A. Motawie, Amany M. Abd Al-Aziz, Abbass A. Mourad, Hassan M. Salama, Solaf Kamel, Eman M. Hassan, Neveen A. Helmy, Gamila S. El-saeed, Eman A. Elghoroury

**Affiliations:** 1 Pediatrics Department, National Research Centre, Dokki, Cairo, 12622 Egypt; 2 Clinical and Chemical Pathology Department, National Research Centre, Dokki, Cairo, 12622 Egypt; 3 Medical Biochemistry Department, National Research Centre, Dokki, Cairo, 12622 Egypt

**Keywords:** Iron deficiency, Hepcidin, TMPRSS6 gene SNP, Minor allele frequency

## Abstract

Several studies have shown association of single nucleotide polymorphisms (SNPs) of hepcidin regulatory pathways genes with impaired iron status. The most common is in the *TMPRSS6* gene. In Africa, very few studies have been reported. We aimed to investigate the correlation between the common SNPs in the transmembrane protease, serine 6 (TMPRSS6) gene and iron indicators in a sample of Egyptian children for identifying the suitable candidate for iron supplementation.

**Patients and methods** One hundred and sixty children aged 5–13 years were included & classified into iron deficient, iron deficient anemia and normal healthy controls. All were subjected to assessment of serum iron, serum ferritin, total iron binding capacity, complete blood count, reticulocyte count, serum soluble transferrin receptor and serum hepcidin. Molecular study of TMPRSS6 genotyping polymorphisms (rs4820268, rs855791 and rs11704654) were also evaluated.

**Results** There was an association of iron deficiency with AG of rs855791 SNP, (*P* = 0.01). The minor allele frequency for included children were 0.43, 0.45 & 0.17 for rs4820268, rs855791 & rs11704654 respectively. Genotype GG of rs4820268 expressed the highest hepcidin gene expression fold, the lowest serum ferroportin & iron store compared to AA and AG genotypes (*p* = 0.05, *p* = 0.05, *p* = 0.03 respectively). GG of rs855791 had lower serum ferritin than AA (*p* = 0.04), lowest iron store & highest serum hepcidin compared to AA and AG genotypes (*p* = 0.04, *p* = 0.01 respectively). Children having CC of rs11704654 had lower level of hemoglobin, serum ferritin and serum hepcidin compared with CT genotype (*p* = 0.01, *p* = 0.01, *p* = 0.02) respectively.

**Conclusion** Possible contribution of SNPs (rs855791, rs4820268 and rs11704654) to low iron status.

**Supplementary Information:**

The online version contains supplementary material available at 10.1186/s12887-024-04573-w.

## Introduction

The worldwide prevalence of iron deficiency anemia (IDA) is about 24.8% of the whole population, with the highest prevalence (47.4%) among children less than five-years of age [1]. Iron deficiency anemia had been known previously, to be combined with environmental and or dietary factors like infections, however, higher prevalence of IDA is still there despite the intensive iron supplementation programs for high-risk groups (children and women of childbearing age) [2]. Also, iron therapy is ineffective in all cases of IDA [3]. This raised the queries about other factors controlling iron status in the human body. The molecular mechanisms modulating iron metabolism and discovery of hepcidin have raised new insights into iron regulation pathways in the human body [4]. Various studies have indicated that 20–30% variability in iron concentration is attributed to genetic factors [5].

Hepcidin induces ferroportin (Fpn) internalization and degradation by binding it on the plasma membrane of cells in various tissues, thus blocking the flux of iron into the blood stream [6]. Many genome-wide association (GWA) studies have shown that single nucleotide polymorphisms (SNPs) in genes implicated in pathways of hepcidin regulation are combined with compromised iron status [7–9]. The commonest SNPs associated with decreased iron status are in the transmembrane protease serine 6 (*TMPRSS6)* gene, which encodes the matriptase-2 protein [10]. Transmembrane protease serine6 gene inhibits hepcidin formation, and its compromised function has been combined with improperly elevated hepcidin, that inhibits absorption of iron by the duodenum and iron mobilization from storage areas [8, 11, 12].

Above 50 SNPs among the *TMPRSS6* gene have been identified to be combined with defective iron status. The most described SNPS are rs855791 followed by rs4820268, rs2235321, rs11704654 and rs2235324. All are associated with low iron status [13–15]. Single nucleotide polymorphisms of rs855791, rs4820268 and rs11704654 have the robust effect on indices of red blood cell and iron parameters specifically in the Asian population [8]. In Africa, very few studies identifying genetic risk factors for anemia in children have been reported [16, 17]. Identification of the major drivers of iron deficiency is mandatory for better management strategies. So, we aimed in this work to investigate the correlation between the common SNPs in the *TMPRSS6* gene and iron parameters in a sample of Egyptian children as a genetic supportive approach for identifying the suitable candidate for iron supplementation.

## Patients and methods

Cross sectional study was conducted in the pediatrics clinic, Centre of excellence, National Research Centre, Egypt as a part of the project titled “*Hematological and non-hematological consequences of iron deficiency in children: Genetic study and response to treatment*”, ID: 11010150. One hundred and sixty children aged 5–13 years were included, they were subjected to full history taking with stress on dietetic history, manifestation of iron deficiency (fatigue, leg cramps on climbing stairs, poor school performance, cold intolerance, & reduced resistance to infection) and iron supplementation during last 6 months. Full clinical examination was carried out including anthropometric measurements (height, weight, and BMI) and stress on signs of iron deficiency (as pallor of the mucous membranes, koilonychia, glossy tongue, atrophy of the lingual papillae and angular stomatitis). Laboratory investigations in the form of complete blood count (CBC), serum iron, total iron binding capacity, serum ferritin, reticulocyte count %, HB electrophoresis, C reactive protein (CRP) and stool analysis were performed. Exclusion criteria were concurrent infection (C reactive protein (CRP) > 5), chronic inflammatory diseases (rheumatic disease, inflammatory bowel disease), chronic disease, parasitic infestation, thalassemia traits α & β (*ruled out by reticulocyte count* < *2% and* hemoglobin *(HB) electrophoresis (A*_*2*_ < *4%*), iron supplementation and blood transfusion during the last 6 months. The included children were classified according to the iron status criteria of World Health Organization (WHO), 2001 [18] into:

- Iron deficiency (ID) (*serum ferritin* ≤ *30 mg/l, transferrin saturation* ≤ *16%, mean corpuscular volume (MCV)* ≤ *73 fl, mean corpuscular hemoglobin concentration (MCHC)* ≤ *32 g/dl*).
- Iron deficiency anemia (IDA), *had* iron deficiency (ID) *plus (hemoglobin level* ≤ 11 g/dl (adopted by Egypt Demographic and Health Survey [19]).
- Normal iron status as a control group.

### Laboratory investigations were done as follow

Eight ml of venous blood were withdrawn under aseptic conditions from 10 to 11am (to avoid diurnal variation), 5 ml were collected into EDTA vacutainer tubes (one ml for CBC & reticulocyte count, 1 ml for hepcidin gene expression and 3 ml for genotyping), the other 3 ml were collected into plain tubes, centrifuged and serum samples were stored at -80 °C for the rest of laboratory investigations.

Complete blood count was done using automated hematology analyzer Sysmx XN100 (Sysmex America Inc), reticulocyte count (using automated hematology analyzer Sysmx XN100 (Sysmex America Inc), C-reactive protein (CRP) (using IMMUNOSPEC REFE 29–056) (Bioquote Ltd, UK), serum iron & total iron binding capacity (TIBC) (using Olympus AU400 (Autoanalyzer, Japan), serum ferritin (SF) (Biocheck, Inc, Cat. No. EC-1025), serum soluble transferrin receptor (sTfR) by ELISA (Cat. No: E0281Hu) (BioVendorInc), serum hepcidin by ELISA (Cat No: E1019 Hu) (Bioassay Technology Lab, China) and serum Ferroportin (Fpn) by ELIZA (Cat. No: E4820Hu).

Transferrin saturation % was calculated using the following formula (WHO, 2001) [18]:

$$\mathrm{TS\%}=\mathrm{ serum\ iron }/\mathrm{TIBC }\times 100$$

Body iron store was assessed using the methodology developed by Cook and coworkers as expressed by the following formula (Cook et al.,) [20]:

$$\mathrm{Body\ iron\ store }({\text{mg}}/{\text{kg}}) = [-\mathrm{log\ }(\mathrm{sTfR\ }/\mathrm{\ SF})-2.8229] /0.1207$$

### Molecular study of hepcidin gene expression using reverse transcriptase polymerase chain reaction

Ribonucleic acid (RNA) was extracted using QIAamp RNA blood mini kits (QIAGEN, Hilden, Germany) according to the manufacturer’s instructions. Samples were extracted on the same day. The final RNA concentration was determined using a spectrophotometer (Nanodrop 2000, Therom Fisher, Walthm, USA) and RNA purity was verified by an average A260/ A280 ratio of 1.98 (range, 1.97–2.01). RNA was reverse transcribed to complementary deoxyribonucleic acid (cDNA) using a high-capacity cDNA reverse transcription kit (Applied Biosystems®, Branchburg, New Jersey, USA) in a final volume of 20 µl. Negative control samples were included in each set of reactions. Reactions were incubated at 25 °C for 10 min, followed by 37 °C for 120 min and final denaturation at 85 °C for 5 min. The reaction was carried out in the Bio-Rad thermal cycler (Life Science Research). cDNA was stored at − 20 °C.

#### Real-time polymerase chain reaction

Gene expression of hepcidin was measured using TaqMan® amplification system (Applied Biosystems®, Branchburg, New Jersey, USA). All samples were run in a final reaction volume of 20 μl. The reaction mix was combined using 10 µl TaqMan® Universal PCR Master Mix, 3 µl of cDNA, 6 µl of DNase free water and 1 µl of specific primers and probes 20 × 20 (Applied Biosystems®, Branchburg, New Jersey, USA). Expression of hepcidin gene was normalized using the Glyceraldehyde 3-phosphate dehydrogenase (GADPH) housekeeping gene. The PCR run was carried out using the thermal profile 50 °C for 2 min, 95 °C for 10 min, 40 cycles of 95 °C for 15 s and 60 °C for 1 min on the Rotor -Gene Q – Qiagen -Germany (Ayatollahi et.,) [21].

#### Genotyping procedure

Genomic DNA was extracted from 3 ml whole blood on EDTA by a commercial DNA extraction kit according to manufacturer’s protocol (QIAamp DNA Blood Mini kit, QIAGEN, USA) CAT No.51104. DNA integrity was determined by 1% agarose gel electrophoresis, stained with ethidium bromide, and visualized through GEL documentation (E-Gel- Imager System with UV Light Base, Thermo Scientific). DNA concentration was determined by Nano Drop 2000 Spectrophotometer (Thermo scientific). TMPRSS6 genotyping polymorphisms

**Table:** 

**rs4820268: **CCTACCTTCCTGGCACTGCTCTTC [A/G] TCGCTGCCGTTGAGACAATCAGGCT, **rs855791: **GCGTGGCGTCACCTGGTAGCGATAG [A/G] CCTCGCTGCACAGGTCCTGTGGGAT **rs11704654: **CCTCACAGGCCTTGAACATCCCCTC[C/T] GGCTCCGCTTCCTCGCCATCACCTC	

were performed using the TaqMan genotyping protocol (Applied Biosystems, Foster City, CA, USA). PCR reactions were set up in 20 μl reaction volume including 20–30 ng DNA, 10 μl TaqMan genotyping PCR Master Mix and 1 μl TaqMan SNP genotyping assay. The PCR assay was carried out according to manufacturer's instructions including one step of 10 min at 95 °C followed by 40 cycles of DNA denaturation at 95 °C for 15 s and annealing/extension at 60 °C for 1 min using the Rotor Gene Q real-time PCR (QIAGEN, Germany). Final products were analyzed by Rotor Gene software (Shinta et al.,) [22].

### Statistical analysis

Data were collected, revised, coded, and statistical package for social science (SPSS) version 16, Inc., Chicago, IL, USA was used for analysis. The data was tested for normal distribution. Quantitative data were presented as mean ± SD. Polymorphism of the *TMPRSS6* genotypes were presented as frequency and percentage. The prevalence of genotypes in the studied groups were tested for deviation from the assumptions of Hardy–Weinberg Equilibrium by using the exact Chi-Square test. Genotype and allele frequencies were compared between patients and control group using Chi-Square tests. Odds ratio (OR) with 95% confidence intervals was calculated. Differences in iron status across the genotype classes were assessed by using ANOVA test to determine the effects of individual SNPs on iron biomarkers. Linear regression analyses were conducted between SNPs rs855791, rs4820268, rs11704654, iron status and hematological parameters. *P*-values ≤ 0.05 were considered statistically significant.

## Results

Based upon the criteria of iron deficiency (ID) and iron deficiency anemia (IDA), 76 (47.5%) of the included children had iron deficiency anemia, 44 (27.5%) had iron deficiency and 40 (25%) were healthy children with normal iron status who served as a control group. Demographic and laboratory data of the three groups are presented in Table 1.

**Table 1:** The Demographic and laboratory data of the three studied groups

**Variables**	Group 1(Control) *N* = 40 (mean ± SD)	Group 2 (ID) *N* = 44 (mean ± SD)	Group 3 (IDA) *N* = 76 (mean ± SD)	*P* value
Age (years)	10.9 ± 1.3	10.2 ± 1.2	9.2 ± 1.5	NS
Sex (male/female)	20/20	24/20	40/36	NS
BMI (Kg/m^2^)	17.4 ± 14.5	18.5 ± 1.2	17.2 ± 1.5	NS
Retics (%)	1.3 ± 0.4	1.2 ± 0.5	1.2 ± 0.6	NS
CRP (mg/L)	0.42 ± 0.2	0.4 ± 0.1	0.33 ± 0.2	NS
HB (gm/L)	12.9 ± 0.86	12.2 ± 0.74	9.5 ± 1.8	*P*^*1*^ = 0.001 *P*^*2*^ = 0.001 *P*^*3*^ = 0.8
Hematocrit (%)	36.7 ± 2.6	35.2 ± 2.6	30.1 ± 3.7	*P*^*1*^ = 0.001 *P*^*2*^ = 0.001 *P*^*3*^ = 0.1
MCV (fl)	77.8 ± 2.3	73.6 ± 3.9	66.7 ± 2.6	*P*^*1*^ = 0.001 *P*^*2*^ = 0.001 *P*^*3*^ = 0.2
MCH (pg)	26.8 ± 2.1	26.1 ± 1.6	22.1 ± 3.9	*P*^*1*^ = 0.001 *P*^*2*^ = 0.001 *P*^*3*^ = 0.2
MCHC (g/dl)	34.8 ± 1.5	31.5 ± 1.6	30.7 ± 2.4	NS
Serum Iron (ug/dl)	92.48 ± 5.4	47.7 ± 1.7	41.3 ± 2.9	*P*^*1*^ = 0.001 *P*^*2*^ = 0.6 *P*^*3*^ = 0.03
TIBC (ug/ml)	316.07 ± 34.4	337.7 ± 5.04	351.03 ± 9.2	NS
Transferrin saturation %	29.4 ± 3.85	15.9 ± 2.6	13.4 ± 3.08	*P*^*1*^ = 0.001 *P*^*2*^ = 0.2 *P*^*3*^ = 0.001
Serum Ferritin (ng/ml)	114.75 ± 16.57	27 .43 ± 6.05	20.49 ± 4.86	*P*^*1*^ = 0.001 *P*^*2*^ = 0.9 *P*^*3*^ = 0.001
Serum human soluble transferrin receptor 1(mg/L)	0.9 ± 0.1	0.16 ± 0.01	1.09 ± 0.2	*P*^*1*^ = 0.4 *P*^2^ = 0.001 *P*^*3*^ = 0.002
Serum Ferroportin (ng/ ml)	8.7 ± 1.7	3.04 ± 0.8	5.5 ± 0.8	*P*^*1*^ = 0.05 *P*^*2*^ = 0.2 *P*^*3*^ = 0.006
Serum hepcidin (pg /ml)	363.4 ± 16.3	285.5 ± 15.7	282.8 ± 13.4	NS
Hepcidin gene expression Fold change	2.6 ± 0.3	0.97 ± 0.1	1.9 ± 0.2	*P*^*1*^ = 0. 1 *P*^*2*^ = 0.07 *P*^*3*^ = 0.01
Body iron store (mg/kg)	1.7 ± 0.2	-5.4 ± 0.2	-9.6 ± 0.9	*P*^*1*^ = 0.001 *P*^*2*^ = 0.001 *P*^*3*^ = 0.001

*BMI* Body mass index, *Retics* reticulocyte count, *CRP* C reactive protein, *HB* hemoglobin, *HCT* hematocrit, *MCV* mean corpuscular volume, *MCH* mean corpuscular hemoglobin, *MCHC* mean corpuscular hemoglobin concentration, *TIBC* total iron binding capacity

*P*^*1*^ (group1&3), *P*^*2*^ (Group 2&3), *P*^*3*^ (group 1&2)

The genotype AG of rs4820268, AG of rs855791 and CC of rs11704654 had the highest percentage among all the studied groups as presented in Table 2.

**Table 2:** Genotype polymorphism and minor allele frequency *of TMPRSS6* gene among the studied groups

Genotype polymorphism No (%)	Group 1 (Control) *N* = 40	Group 2 (ID) *N* = 44	Group 3 (IDA) *N* = 76	*P* value
**Polymorphism of rs4820268**				
AA homozygote	11 (27.5%)	16 (36.4%)	24 (31.6)	0.7
AG heterozygote	20 (50%)	20 (45.5%)	40 (52.6%)	0.8
GG homozygote	9 (22.5%)	8 (18.1%)	12 ((15.8)	0.8
**Polymorphism of rs855791**				
AA homozygote	10 (25%)	4 (9.15)	8 (10.5%)	0.06
AG heterozygote	28 (70%)	40 (90.9%)	64 (84.2%)	*P1* = 0.02 *P2* = 0.1 *P3* = 0.001
GG homozygote	2 (5%)	0 (0%)	4 (5.3%)	0.3
**Polymorphism of rs11704654**				
CC homozygote	24 (63.2%)	26 (59.1%)	54 (71.1%)	0.3
CT heterozygote	16 (36.8%)	18 (40.9%)	22 (28.9%)	0.3
**Minor Allele Frequency (MAF)**				
rs4820268 (G)	38 (47.5%)	36 (40.9%)	64 (42.1%)	0.6
rs855791 (G)	32 (40%)	40 (45.5%)	72 (48.4%)	0.5
rs11704654 (T)	16 (20%)	18 (20.4%)	22 (14.5%)	0.4

*P1* = (group1&3), *P2* (Group 2&3), *P3* (group 1&2)

All the SNPs investigated were in Hardy–Weinberg Equilibrium (*P* > 0.05) Mayo, [23].

**Fig. 1:**
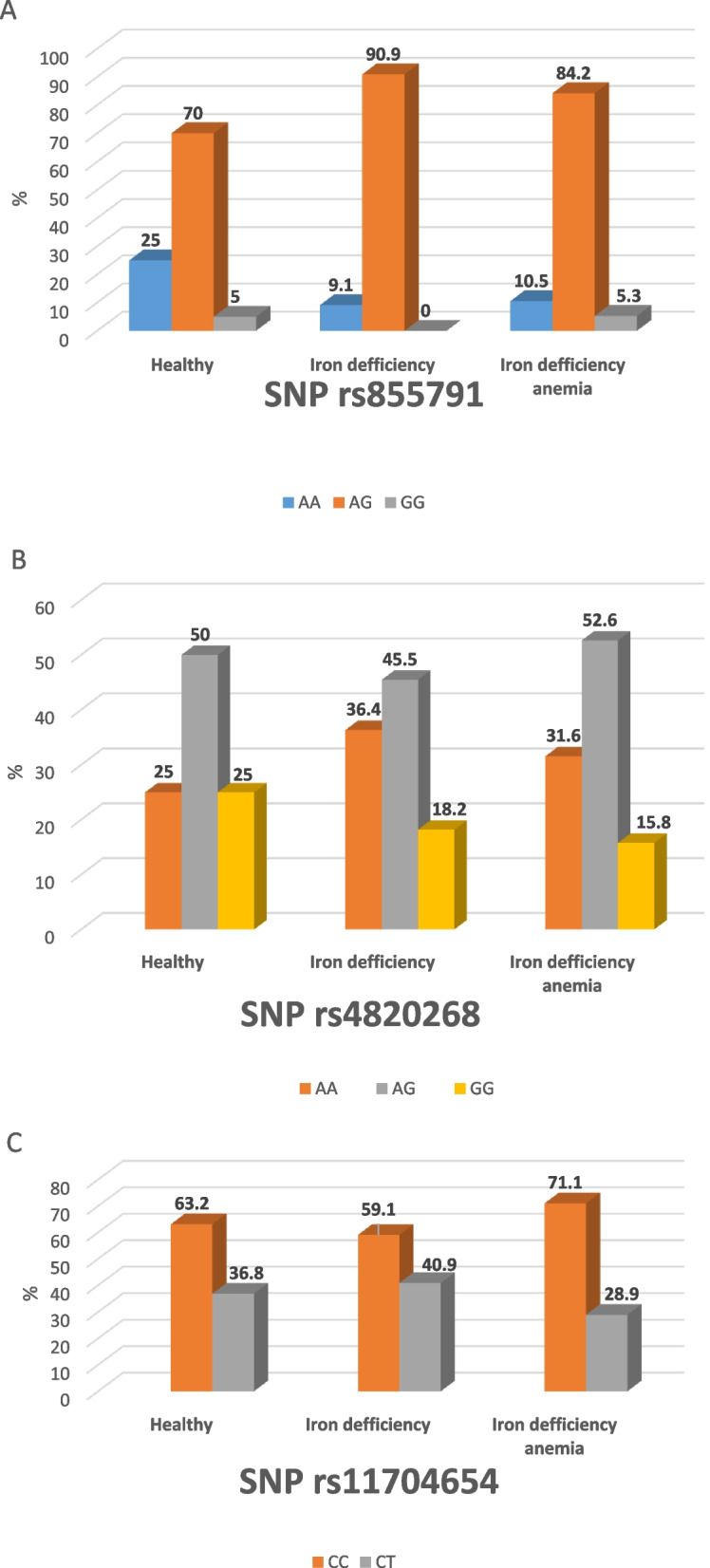
Distribution of SNPs rs855791 (**A**), SNPs rs4820268 (**B**) and SNPs rs11704654 (**C**) among normal iron status (healthy), iron deficiency and iron deficiency anemia groups

Increasing frequency of heterozygote allele genotype AG of rs855791 in iron deficiency and iron deficiency anemia compared to healthy controls (*p* = 0.001 & *p* = 0.02 respectively) was detected as presented in Fig. 1A, while the frequency distribution of allele genotypes of rs4820268 and rs11704654 showed no significant difference (Fig. 1B and C).

By using the odds ratio (OR), an association of iron deficiency with AG genotype of rs855791 was found, Odds Ratio with 95% confidence interval (OR (95% CI) = 3.33 (1.298–8.558), *P* = 0.01 (Table 3).

**Table 3:** Genotype and allele distribution of SNPs in *TMPRSS6* gene among Iron deficiency (ID), iron deficiency anemia (IDA), and normal iron status groups

Variables		Patients (ID & IDA) *N* = 120		Controls *N* = 40		*P* value	OR	RR	95%CI	
		Number	Percent (%)	N	%				Lower	Upper
rs4820268 Genotype	AA	40	33.3%	10	25%	0.4	0.75	0.833	0.318	1.769
	AG	60	50%	20	50%	1				
	GG	20	16.7%	10	25%	0.7	0.833	0.952	0.318	2.184
rs4820268 alleles:	A	140	58.3%	40	50%	1				
	G	100	41.7%	40	50%	0.19	1.4	1.08	0.842	2.326
rs855791 Genotype	AA	12	10%	10	25%	1				
	AG	104	86.7%	28	70%	0.01	3.33	2.68	1.298	8.558
	GG	4	3.3%	2	5%	0.6	0.5	0.6	0.087	2.88
rs855791 alleles	A	128	53.3%	48	60%	1				
	G	112	46.7%	32	40%	0.3	.762	.935	.456	1.274
rs11704654 Genotype	CC	80	66.7%	24	60%	1				
	CT	40	33.3%	16	40%	0.4	.75	.9	.359	1.568
rs11704654 alleles	C	200	83.3%	64	80%	1				
	T	40	16.7%	16	20%	0.5	1.250	1.06	0.656	2

*N* Number, *OR* odds ratio, *RR* relative risk, *95%CI* 95% confidence interval.* P* < 0.05: significant.* P* < 0.01: highly significant

The minor alleles frequency for all children (patients and controls) was found to be 0.43, 0.45 and 0.17 for rs4820268, rs855791 and rs11704654 respectively (Table 4).

**Table 4:** Variation in minor allele frequency among our results compared to African, American, and European populations (*in the 1000 Genomes Project*) [24]

		Minor alleles frequency (percentage %)			
SNP	Minor/Major	Our results	African populations	American populations	European populations
rs4820268	G/A	0.43	0.28	0.53	0.42
rs855791	G/A	0.45	0.10	0.49	0.39
rs11704654	T/C	0.17	0.15	0.11	0.19

Children having minor homozygote allele GG genotype of rs4820268 had the highest hepcidin gene expression fold, the lowest serum ferroportin and iron store compared to those having AA and AG genotypes (*p* = 0.05, *p* = 0.05, *p* = 0.03 respectively). Children having minor homozygote allele GG genotypes of rs855791 had lower serum ferritin level compared to AA (*p* = 0.04). They had the highest level of serum hepcidin and lowest iron store compared to AA and AG genotypes (*p* = 0.04, *p* = 0.01 respectively). Children having major homozygote allele CC genotype of rs11704654 had lower level of hemoglobin, serum ferritin and serum hepcidin compared with CT genotype (*p* = 0.01, *p* = 0.01, *p* = 0.01, *p* = 0.01, *p* = 0.02 respectively), (Table 5).

**Table 5:** Characteristics of iron status of the studied children stratified by the studied genotypes

	rs4820268				rs855791				rs11704654		
Variables	AA	AG	GG	P	AA	AG	GG	P	CC	CT	P
Number	50	80	30	0.1	22	132	6		104	56	
HB (gm/dl)	11.6 ± 2	11 ± 1.4	10.8 ± 2.2	0.8	11.15 ± 0.6	11.17 ± 0.1	10.8 ± 0.4	0.9	10.8 ± 0.2	11.6 ± 0.2	0.01
S. iron (ug/dl)	57.7 ± 4.3	56.3 ± 3.8	53.2 ± 5.3	0.3	50.1 ± 4.2	57.4 ± 2.7	49.3 ± 5.7	0.5	53.3 ± 2.8	57.3 ± 4.1	0.4
S. ferritin (ng/dl)	34.4 ± 3	39.6 ± 3.7	30.1 ± 4.1	0.3	45.3 ± 53.2	34.9 ± 2.6	34.8 ± 7.3	0.04	31.2 ± 2.8	42 ± 4.5	0.01
S. sTfR1 (mg/L)	0.73 ± 0.15	0.53 ± .15	0.94 ± 0.3	0.05	0.13 ± 0.01	0.7 ± 0.1	1.6 ± 0.01	0.1	0.8 ± 0.14	0.5 ± 0.11	0.2
S. Ferroportin (ng/ml)	6.4 ± 1.4	6.4 ± 0.8	2.5 ± 0.8	0.7	4.8 ± 1.9	5.5 ± 0.7	6.8 ± 1.3	0.7	5.6 ± 0.8	5.2 ± 0.9	0.7
S. hepcidin (pg/ml)	345.3 ± 36.7	290.9 ± 30.3	277.3 ± 23.9	0.05	211.4 ± 36.6	287.75 ± 30.2	569.2 ± 118.4	0.01	247.3 ± 29.5	375 ± 49.9	0.02
Hepcidin gene expression fold	0.87 ± 0.15	1.98 ± 0.23	2 ± 0.35	0.03	1.9 ± 0.4	1.7 ± 0.2	2.7 ± 0.6	0.3	1.9 ± 0.2	1.6 ± 0.3	0.4
Body iron store (mg/kg)	-6.6 ± 0.8	-5.3 ± 0.7	-9.4 ± 1.2	0.1	-4.3 ± 1.8	-6.6 ± 0.5	-12.3 ± 1.7	0.04	-7.3 ± 0.7	-5.6 ± 0.8	0.1

Data expressed as mean ± SD, *P* < 0.05: significant, *P* < 0.01: highly significant. *S* serum

By linear regression analysis, and after adjustment of age and BMI, SNPs of rs4820268 explained 4% of the change in HB level (*r*^*2*^ = 0.04, *P* = 0.04), 5% of the variation in serum ferroportin concentration (*r*^*2*^ = 0.05, *P* = 0.04). Single nucleotide polymorphisms of rs855791 explained 8.5% of the variation in iron store (*r*^*2* =^0.085, *P* = 0.04) and 8.2% of the change in serum hepcidin (*r*^*2*^ = 0.082, *P* = 0.009). Single nucleotide polymorphisms of rs11704654 explained 4.5% of the change in HB level (*r*^*2* =^0.045, *P* = 0.01), and 6.5% of the change in serum hepcidin (*r*^*2*^ = 0.065, *P* = 0.02).

The impact of gender on genes associations was not detectable, so sex was not considered in the study (data was represented as Supplementary table).

## Discussion

Single nucleotide polymorphisms of rs855791 of TMPRSS6 gene has been widely described to affect iron indices and to be correlated with the risk of IDA in Europeans [15] and Asians [14, 25]. Our study showed that iron deficiency and iron deficiency anemia have significantly higher heterozygote allele’s genotype AG of rs855791 compared to control that agreed with Elmahdy et al., [26]. Shinta et al., [22] found that both iron deficiency and iron deficiency anemia were associated with minor homozygote allele of rs855791 genotype. On the other hand, Momodou et al., [27] didn’t find any association with iron status biomarkers.

Among the Asian, African, and European populations there is a significant divergence in the minor allele frequency distribution of SNPs [8, 17, 28]. The minor allele frequency (MAF) of SNP rs855791, rs4820268 and rs11704654 found to be less in African than Asian and European populations [17]. The difference may be attributed to the selective role of certain environmental conditions that alter the frequency of the genetic variants among populations [29]. Our study showed that MAF(G) of rs855791, (G) of rs4820268 and (T) of rs11704654 were 0.43, 0.45 and 0.17 respectively that were comparable to the European ancestry population but not to the African. This similarity may be due to mix of genes between Egyptians and Europeans by marriage in the past times where Europeans existed in our country for different purposes like trading, working or wars. Theoretically these differences in MAFs among populations could result in divergencies in the incidence of iron deficiency outcome [30]. However, the minor alleles for SNPs of certain population may be the major alleles in other populations [17]. For example, Shinta et al., [22] found that the minor allele of rs855791 genotype was [A] while it is the major allele in our work and [G] is our minor allele.

Disparities in the frequencies of minor risk alleles and linkage disequilibrium forms might explain the limited association of results between European, Asian, and African populations. So, investigating population-specific genetic variants, is recommended [27]. Wanjiku et al. [17], called for population-specific genome studies rather than inferring genetic data across populations.

Our study revealed that the minor homozygote allele genotype (GG) of rs4820268 is associated with the highest hepcidin gene expression fold, the lowest serum ferroprotein and iron store. Also, the minor homozygote allele genotype (GG) of rs855791 is associated with the lowest iron store, highest level of serum hepcidin and low serum ferritin. Higher hepcidin level reduces ferroprotein expression, inhibits iron absorption by the duodenum and iron mobilization from storage (Benyamin et al., Chambers et al.) [7, 8]. Thus, minor homozygote allele genotype (GG) of rs4820268 and rs855791 might be associated with Iron-refractory iron deficiency anemia (IRIDA) that might not respond to oral iron supplement. Higher levels of serum hepcidin were found to confirm the diagnosis of (IRIDA) [31, 32]. However, hepcidin assessment has no inter-laboratory comparison of assays and no standardization of units and reference ranges, that could facilitate the clinical use of a hepcidin [33, 34]. So, genetic assessment could be more supportive.

Shinta et al., [22] reported a lower serum ferritin in minor homozygote allele genotype of both rs855791 and rs4820268 even after controlling iron intake. They added that, they are naturally diminished by up to 10% of the total body iron store when compared to the major homozygote. A lower hemoglobin level, serum ferritin and serum hepcidin were detected in our studied children with homozygote major allele CC genotype of rs11704654 that agreed with results of Delbini, et al., [15]. Iron deficiency diminishes hepcidin expression and permits FPN1 to be available for transporting iron from intestine and tissues through the blood stream [35, 36]). Accordingly, this group may benefit from oral iron supplementation.

In a linear regression model, SNPs of rs4820268 explained 4% of the change in HB level, 5% of the variation in serum ferroprotein concentrations while SNPs of rs855791 explained 8.5% of the change in iron store and 8.2% of the change in serum hepcidin. Our results agreed with Tanaka et al., and Shinta et al. [5, 22], who found significant association of SNPs rs855791 and rs4820268 with iron status. Wanjiku et al., [17] could not discover any effect of these two SNPs on iron- status.

The relation of the three SNPs of our results with iron status and hepcidin were comparable to studies by others [7, 8, 15, 25, 28, 37]. On other hand, Momodou et al., [27] did not identify any TMPRSS6 SNP association with hepcidin concentration or iron status. Wanjiku et al., [17] attributed this to heterogeneity in genetic makeup and environmental disparities between populations that alter the frequency genetic variants.

Our study, urges for sub-population specific strategies to address iron deficiency & who can benefit from iron therapy to maintain normal erythropoiesis. Also, validation and discovery of further genetic variants correlated with iron status to clarify new processes and pathways that modify iron status, propounding better understanding into ID/IDA.

## Limitation of this study

The children were recruited from a single center; it could be better to be a multicenter study which could not be fulfilled due to budget limitation.

## Conclusion

Our work highlights the possible contribution of TMPRSS6 gene SNPs to low iron status in Egyptian children & who can benefit from oral iron therapy. A longitudinal study of the interaction between genetic variants and iron biomarkers on a larger sample size from different environmental sectors is recommended that will be arranged in near future study.

### Supplementary Information

**Additional file 1: Supplementary table.** Genotype stratified by sex of all studied children.

## Data Availability

All generated data is present in the manuscript.

## Abbreviations

GWAS: Genome-wide association studies
SNPs: Single nucleotide polymorphisms
IRIDA: Iron-refractory iron deficiency anemia
*TMPRSS6*: Transmembrane protease serine 6
*TF*: Transferrin
MAF: Minor Allele Frequency
MCV: Mean corpuscular volume
CBC: Complete blood count
RBC: Red blood cells
TSAT: Transferrin saturation
sTfR: Soluble transferrin receptor
TIBC: Total iron binding capacity
BMI: Body mass index
CRP: C reactive protein,
HCT: Hematocrit
