# Supplementary material for: The association of TMPRSS6 gene polymorphism with iron status in Egyptian children (a pilot study)
Source: BMC Pediatr. 2024 Feb 10;24:105. doi: 10.1186/s12887-024-04573-w (PMC10858485; doi:10.1186/s12887-024-04573-w)
Supplement: Supplementary file 1 — Additional file 1: Supplementary table. Genotype stratified by sex of all studied children. [file 12887_2024_4573_MOESM1_ESM.docx]

**Supplementary table: Genotype stratified by sex of all studied children.**

| Variables | **Polymorphism of rs4820268** | | | **Polymorphism of rs855791** | | | **Polymorphism of** r**s11704654** | |
| --- | --- | --- | --- | --- | --- | --- | --- | --- |
|  | AA | AG | GG | AA | AG | GG | CC | CT |
| Males  Number (84)  Percent (%) | 28  33.3% | 40  47.6% | 16  19.1% | 11  13.1% | 69  82.1 | 4  4.8% | 52  61.9% | 32  38.1% |
| Females Number (76)  Percent (%) | 22  28.9% | 40  52.6% | 14  18.4% | 11  14.8% | 63  82.7% | 2  2.5% | 52  68.4% | 24  31.6% |
| P Value | 0.7 | | | 0.8 | | | 0.3 | |

p>0.05 is not significant.
